# Chinese diaspora caregivers’ experiences in dementia care in high-income countries: A systematic review

**DOI:** 10.1177/14713012231169830

**Published:** 2023-04-18

**Authors:** Yujing Zhang, Lily Xiao, Jing Wang

**Affiliations:** College of Nursing and Health Sciences, 1065 Flinders University, Bedford Park, SA, Australia; Faculty of Nursing, Health Science Center, 12480 Xi’an Jiaotong University, Xi'an, China

**Keywords:** Chinese diaspora, dementia, family caregiver, experience, systematic review

## Abstract

**Background:**

Chinese diaspora caregivers in high-income countries make up a large proportion of the ethnic population and usually experience significant challenges in the care of their family members with dementia.

**Aim:**

The aims of this systematic review were to gain deep insights into Chinese diaspora caregivers’ experiences and factors contributing to their experiences in the care of family members living with dementia in high-income countries.

**Methods:**

The Joanna Briggs Institute (JBI) meta-aggregative approach to qualitative studies was applied to this systematic review. The review also followed the PRISMA guidelines and was informed by the Life Course Theory. Six English databases were searched between August 2020 and September 2020.

**Results:**

In total, 330 articles were screened and 16 were included in the review. The number of caregivers included in these studies was 365 across four countries. Four synthesised findings with sub-themes were identified from studies reviewed. These synthesised findings were described as: (1) motivations to take on the caregiving role; (2) receiving limited dementia care education; (3) factors affecting access and use of care services; and (4) experiencing multifaced challenges.

**Conclusion:**

Dementia care policies need to address disparities between caregiver support for the mainstream group and Chinese diaspora caregivers. Dementia education and care services need to consider the positive impact that filial piety and Confucianism have on Chinese diaspora caregivers and empower them to use their strengths. Dementia care services need to be culturally adapted to meet this care group’s needs, preferences and expectations.

## Introduction

The incidence of dementia is rising and approximately 10 million new cases are reported every year globally (World Health Organization, 2020). China has the largest population living with dementia (Jia et al., 2020; The World Bank, 2020). China also possesses a large population that have emigrated to high-income countries such as the United States (US), Canada, Australia, Singapore and the United Kingdom (UK) (Statista, 2022). Studies have revealed that Chinese diaspora face unique challenges in the care of family members living with dementia in high-income countries due to various factors ranging from the influence of Chinese cultural norms and dementia stigma to inadequate health and social care services that hinder use of dementia care services (Baghirathan et al., 2020; Liang et al., 2021). The term ‘Chinese diaspora’ in this paper means Chinese descent who live outside Mainland China, Hong Kong, Taiwan and Macau (Wong & Tan, 2018). In a population-based survey conducted in the US, Chinese Americans showed a higher proportion of perceived embarrassment for family members with dementia than other Asian ethnic groups (Liang et al., 2021). The finding was confirmed by a systematic review (Nguyen & Li, 2020). These findings indicate different caregiver experiences exist between migrant groups living in high-income countries. Therefore, viewing migrants as one group when conducting dementia research may prevent policy makers and service providers from developing acceptable and effective culturally adapted interventions (Hall et al., 2016; Spanhel et al., 2021). Analysing caregivers’ experiences within each ethnic group is an effective approach to identifying factors affecting practices and perspectives in order to achieve person-centred dementia care (American Geriatrics Society Expert Panel on Person-Centered Care, 2016).

Confucianism, a Chinese philosophy, strong influences Chinese diaspora caregivers’ motivation to undertake the caregiving role (Liu et al., 2020). This philosophy educates Chinese people to respect and look after their parents (filial piety), care for their spouse and support extended family members in order to achieve peace and harmony in the family (Yiu et al., 2020). Filial piety is explained as ‘honour and devotion to one’s parents …[and] implies that adult children have a responsibility to sacrifice individual physical, financial and social interests for the benefit of their parents or family’ (Greenwood & Smith, 2019, p. 15). Filial piety motivates adult-child caregivers to undertake and sustain their caregiving role despite difficulties (Guo et al., 2019). Chinese diaspora caregivers who embrace Confucianism usually view caring for family members with dementia as their responsibility. Therefore, they may feel guilty if they use formal care services which has implications for their emotional wellbeing (Baghirathan et al., 2020; Brijnath et al., 2021). A large cross-sectional study conducted in the US revealed that Chinese diaspora caregivers held different levels of filial piety according to the extent of their adaptation to the cultural values of the host country (Guo et al., 2019). The finding indicates the strong influence of migration processes on Chinese diaspora caregivers. Therefore, dementia care services for this group of caregivers needs to consider their experiences through a life course perspective (Liu et al., 2020).

Studies on Chinese diaspora caregivers identified that dementia associated self-stigma was highly prevalent and a main factor that delayed them from seeking help outside of the family (Liang et al., 2021; Nguyen & Li, 2020). Caregivers’ self-stigma is defined as the internalisation of dementia related public stigma (Nguyen & Li, 2020). The consequences of self-stigma included hiding dementia, disconnecting with friends and communities, and high caregiver stress and burden (Baghirathan et al., 2020; Kenning et al., 2017). Caregivers’ self-stigma was associated with poor quality of life (Nguyen & Li, 2020) and low usage of dementia care services (Liang et al., 2021). The high level of self-stigma among Chinese diaspora caregivers was attributed to limited English proficiency, low education levels, limited knowledge and misconceptions about dementia (Baghirathan et al., 2020; Brijnath et al., 2021). This underscores the need for ethno-specific services in dementia education and support for this group of caregivers.

Chinese diaspora caregivers usually experienced difficulties accessing appropriate dementia care services in high-income countries (Baghirathan et al., 2020; Brijnath et al., 2021). The language barrier was constantly mentioned as a contributing factor that hindered them from searching for information about dementia care services and receiving dementia caregiver education (Ma & Saw, 2020). Studies in the US and UK identified that Chinese welfare/charity organisations were usually the first line of contact for this caregiver group (Baghirathan et al., 2020; Ma & Saw, 2020). However, their needs were viewed as partially met via these sources due to the lack of dementia-specific training for staff and inadequate resources in these organisations to provide caregiver support (Baghirathan et al., 2020; Ma & Saw, 2020).

Although many studies reported dementia caregiver experiences and challenges in dementia care, fewer systematic reviews have been conducted to synthesise findings from Chinese diaspora caregivers in high-income counties. This gap in the literature will negatively affect evidence-based and tailored support for Chinese diaspora caregivers in high-income counties. The present systematic review addresses the gap in the literature by generating new understandings of Chinese diaspora caregivers’ experiences and factors contributing to their experiences in dementia care in high-income countries in a global context. Moreover, the life course theory developed by Elder et al. (2003) has been applied to this review to enhance the understanding of Chinese diaspora caregivers’ experience located in their life course.

### Theoretical Framework

Disparities in supporting caregivers of people with dementia are well recognised in high-income countries (World Health Organization, 2021). Eliminating these disparities requires an in-depth understanding of socially, culturally and historically constructed factors affecting caregivers’ practices. This review is informed by the Life Course Theory that focuses on these factors (Elder et al., 2003). This theory was applied by Liu et al.’s (2020) in exploring dementia caregivers’ challenges and resilience previously. This theory described five interrelated principles that enable researchers to explore the impact of social-historical conditions people experience in their lives on their developmental experiences, human agency and relations to others. We interpret the five principles and their applications in the context of this review as follows.

First, ‘the principle of life-span development’ views human development on a continuum and considers people’s previous experiences to have a profound impact on their current and future development. The application of this principle is that Chinese diaspora caregivers’ experiences in dementia care need to be located into their life course, for example the influence of Chinese cultural norms (Confucianism) developed in their home country and the influence of acculturation developed in the host country on caregivers’ behaviour. Second, ‘the principle of agency’ values human beings as an active being who makes decisions and choices based on rationality despite constraints from historical and social circumstances. The application of this principle is that Chinese diaspora caregivers choose, or refute, care services based on their judgement of the relevance, usefulness and acceptability. Third, ‘the principle of time and place’ describes that human experiences are shaped by the historical context. This means that Chinese diaspora caregivers’ difficulties in accessing dementia care services are shaped by the design of these care services for the mainstream culture in a historical context. Fourth, ‘the principle of timing’ indicates that lived experience varies based on the timing within an individual’s life. This principle indicates that Chinese spouse and adult caregivers may have different experiences in dementia care due to age variations. Finally, ‘the principle of linked lives’ espouses that an individual’s lived experiences shape and are shaped by others related to them. The application of this principle is that Chinese diaspora caregivers’ experiences are influenced by the care recipient, other family members, peers and service providers.

### Aims of the Study

The aims of this systematic review were to gain deep insights into Chinese diaspora caregivers’ experiences and factors contributing to their experiences in the care of family members living with dementia in high-income countries.

## Method

The Joanna Briggs Institute (JBI) meta-aggregative approach was applied to the synthesis of qualitative studies (Joanna Briggs Institute, 2020). The review also complied with the Preferred Reporting Items for Systematic Reviews and Meta-Analyses (PRISMA) guidelines. The review protocol was registered on the PROSPERO Web site (registration number: CRD42020205511).

### Search Strategy

We applied the *population*, phenomenon of *interest* and *context* (PICo) framework suggested by Stern and colleagues to form keywords: ‘family caregiver’, ‘experience’, ‘dementia’ and ‘Chinese’ (Stern et al., 2014). Synonyms of each keyword were then analysed to create a logic grid to capture relevant studies (Lockwood et al., 2020) (see Online Appendix I). We searched six English databases between August 2020 and September 2020 including Cumulative Index to Nursing and Allied Health Literature (CINAHL), Emcare, Medical Literature Analysis and Retrieval System Online (MEDLINE), ProQuest, Scopus, and Web of Science. No limited publication dates were used during the search for valid studies. The first author searched each database, and an expert librarian examined each search process. The search processes for each database are shown in Online Appendix II.

### Inclusion Criteria

Inclusion criteria for this review were: (1) studies that used a qualitative design or a mixed-methods design with a qualitative component; (2) adult family caregivers aged 18 years or older who cared for a person living with dementia; (3) the phenomena of interest included experiences, perceptions, feelings and emotions of Chinese family caregivers; (4) the family caregivers are Chinese diaspore; (5) the context of included studies was the home setting in high-income countries as defined by the World Bank (World Bank, 2021); (6) published in English language.

### Search Outcome

**Figure 1.**
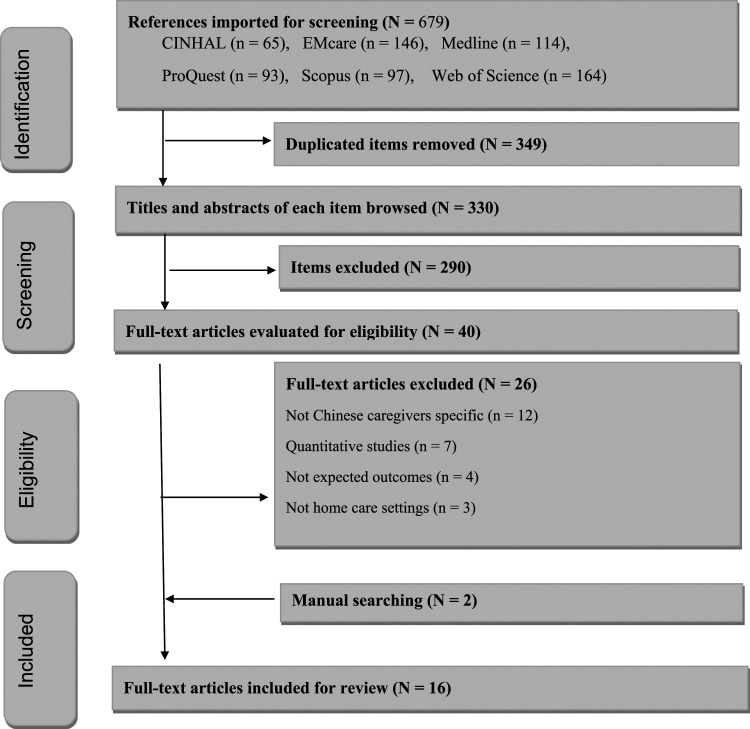
PRISMA chart: Searching and screening process.

In total, 679 articles were identified from database searches. All were exported into EndNote (Version X9.3.3) and imported into Covidence software for screening. After the removal of 349 duplicate studies, 330 articles were screened by title and abstract. A total of 290 were subsequently excluded based on the inclusion criteria. We further reviewed the full text of the remaining 40 articles and excluded 26 articles. We undertook a manual search of reference lists for the 14 selected articles and identified a further two relevant articles. In total, 16 articles were selected for critical appraisal (see Figure 1). Three authors independently participated in each stage of the selection. Each article was screened and reviewed by two members of the review team independently (YZ and JW) and one reviewer resolved any conflicts (LX).

### Quality Appraisal

This review adopted the JBI Qualitative Critical Appraisal Checklist (Version 2017) to assess the selected studies (Joanna Briggs Institute, 2017). Two reviewers undertook a critical review for each article independently and any disagreements were discussed in regular team meetings. The review team agreed to use 8 out of 10 on the JBI appraisal tool as the cut-off point based on their previous experience. All 16 selected article passed the critical review, and detailed appraisal information is outlined in Table 1.

**Table 1.** Critical appraisal for included studies.

Author, year	Q1.	Q2.	Q3.	Q4.	Q5.	Q6.	Q7.	Q8.	Q9.	Q10.
Koo et al., 2020	Yes	Yes	Yes	Yes	Yes	Yes	Yes	Yes	Yes	Yes
Liu et al., 2020	Unclear	Yes	Yes	Yes	Yes	Yes	Yes	Yes	No	Yes
Tan et al., 2020	Yes	Yes	Yes	Yes	Yes	Unclear	Unclear	Yes	Yes	Yes
Chan et al., 2019	Yes	Yes	Yes	Yes	Yes	Yes	Unclear	Yes	Yes	Yes
Lun 2019	Unclear	Yes	Yes	Yes	Yes	Yes	Yes	Yes	Yes	Yes
Tuomola et al., 2016	Yes	Yes	Yes	Yes	Yes	Yes	Yes	Yes	Yes	Yes
Caldwell et al., 2014	Unclear	Yes	Yes	Yes	Yes	Unclear	Yes	Yes	Yes	Yes
Sun et al., 2014	Unclear	Yes	Yes	Yes	Yes	Yes	Yes	Yes	Yes	Yes
Vaingankar et al., 2013	Unclear	Yes	Yes	Yes	Yes	Yes	No	Yes	Yes	Yes
Xiao et al., 2013	Yes	Yes	Yes	Yes	Yes	Yes	Yes	Yes	Yes	Yes
Koehn et al., 2012	Yes	Yes	Yes	Yes	Yes	No	Yes	Yes	Yes	Yes
Boughtwood et al., 2011	Unclear	Yes	Yes	Yes	Yes	Yes	Yes	Yes	Yes	Yes
Netto et al., 2009	Yes	Yes	Yes	Yes	Yes	Unclear	Unclear	Yes	Yes	Yes
Zhan, 2004	Yes	Yes	Yes	Yes	Yes	Unclear	Yes	Yes	No	Yes
Ho et al., 2003	Unclear	Yes	Yes	Yes	Yes	Yes	Yes	Yes	Yes	Yes
Tan et al., 2001	Unclear	Yes	Yes	Yes	Yes	Yes	No	Yes	Yes	Yes

Q1. Is there congruity between the stated philosophical perspective and the research methodology?

Q2. Is there congruity between the research methodology and the research question or objectives?

Q3. Is there congruity between the research methodology and the methods used to collect data?

Q4. Is there congruity between the research methodology and the representation and analysis of data?

Q5. Is there congruity between the research methodology and the interpretation of results?

Q6. Is there a statement locating the researcher culturally or theoretically?

Q7. Is the influence of the researcher on the research, and vice versa, addressed?

Q8. Are participants, and their voices are adequately represented?

Q9. Is the research ethical according to current criteria or, for recent studies, and is their evidence of ethical approval by an appropriate body?

Q10. Do the conclusions drawn in the research report flow from the analysis, or interpretation, of the data?

### Data Abstraction

The JBI Qualitative Assessment and Review Instrument (JBI-QARI) tools was used to extract data for analysis. These tools include summarising the characteristic of included studies and extraction of findings with illustrations (Lockwood et al., 2020, p. 55). To ensure that the extracted findings and interpretations were consistent with the intended meanings of primary authors, the reviewers carefully analysed the extraction of findings against the illustrations based on the degree of support at the three levels of credibility set out in the JBI review manual: unequivocal, credible and not supported (Lockwood et al., 2020). Results from data extractions and credibility assessments are presented in Online Appendix III.

### Data Synthesis

Based on the JBI meta-aggregated approach, a three-step process for data synthesis was conducted (Hannes & Lockwood, 2011; Lockwood et al., 2020). First, the reviewers extracted findings from the original studies by primary researchers. Then, based on similarities among these findings, the reviewers assigned different categories. Finally, the reviewers further synthesised categories into themes based on their relationships and relevance to the review aims. The three reviewers conducted regular meetings to reach agreement on the final findings. Disagreements were resolved through discussions. Online Appendix IV presents detailed results of the meta-synthesis.

## Results

### Characteristics of the Studies Reviewed

Of the 16 articles included in the review, six were conducted in Singapore, four in the US, four in Australia and two in Canada. These articles reported findings from 365 caregivers including 252 adult-child caregivers, 87 spouse caregivers and 26 relative caregivers. Of those caregivers, 263 were female (72.1%) and 102 were male. Their ages ranged from 18 to 93 years, and the mean age was 55.5 years. Five studies included multiple ethnic groups, but only data for Chinese participants was extracted from these (Boughtwood et al., 2011; Caldwell et al., 2014; Chan et al., 2019; Vaingankar et al., 2013, Xiao et al., 2013). Eleven studies were conducted exclusively with Chinese participants. Detailed characteristics of all 16 studies are summarised in Table 2.

**Table 2.** Characteristic of included studies.

Author, Year	Methodology/Method	Phenomena of interest	Setting/Geographical	Participants	Data analysis	Authors conclusion
Koo et al., 2020	Longitudinal qualitative study	To explore caregivers’ experience of taking care of family member living with dementia	Home care	9 participants from 5 families (4 daughters, 2 sons, 2 grandsons, and 1 son-in-law)	Narrative analysis	The intergenerational family care of people with dementia can show a positive aspect in family bonding
	Semi-structured biographical interview and digital photographs		Singapore			
Liu et al., 2020	Hybrid grounded theory model Qualitative study	Under the migration and sociocultural background, to explore the family caregivers’ challenge and experiences of caring people living with dementia	Home care	27 caregivers (14 adult child caregivers; 12 spouse caregivers; 1 sibling)	Three-step hybrid model	The themes that found in the study demonstrate caregivers’ care needs and experiences, and it has an implication of practice in the future
	Semi-structured face-to-face interview		United State, New York			
Tan et al., 2020	Thematic analysis Qualitative study	To explore the difficulties and barriers of making decision while caregivers taking care of people with dementia	Home care	14 participants (4 daughters, 7 sons, 2 spouses, and 1 family relative)	Thematic analysis	The family caregivers lack of knowledge and understanding of dementia and making decision, and there is a need of enhancing awareness of dementia in order to inform them making decision
	Interview		Singapore			
Chan et al., 2019	Descriptive phenomenological approach Qualitative study	To explore the Asian family caregivers’ experiences of caring of people with dementia	Home care	16 family caregivers (2 wives, 9 daughters, 3 sons and 2 family relatives)	Colaizzi’s 7-step approach	Family caregivers’ feelings are different at each stage while taking care of people with dementia. Also, it is crucial to provide information regarding dementia for family caregivers
	Semi-structured face-to-face interview		Singapore			
Lun 2019	Qualitative study	To develop Chinese American caregivers’ perceptions and care needs of caring for family member with dementia	Home care	4 caregivers (2 spouses, and 2 daughters)	Content analysis	Reflecting caregivers’ feelings and increasing understanding on caregivers’ stresses. Also, the results have a better implication for health workers and community nurses
	In-depth Interview		United States, New York			
Tuomola et al., 2016	Interpretative phenomenological analysis Qualitative study	To examine caregivers’ experience of taking care of people with dementia and sense of self	Home care	6 family caregivers (6 wives)	Interpretative phenomenological analysis	The family caregivers’ sense of self will be influenced by different lived experiences regarding supporting dementia care, and the findings imply that it also be influenced by confucian values
	Semi-structured interview		Singapore			
Caldwell et al., 2014	Qualitative study	To explore caregivers’ experiences that make decision of placing family member living with dementia into nursing home	Home care and nursing home	27 family caregivers (20 Chinese,7 English speaking background)	Thematic analyses	There is a need for providing home care service for caregivers in the care of people with dementia, and also the waiting time of placing people with dementia in nursing home should be decreased
	Semi-structured interview design		Australia, Sydney			
Sun et al., 2014	Qualitative study	Identify the service barries from the Chinese American family caregivers’ and professional’ perspectives	Home care	6 professionals and 6 caregivers (2 wives, 1 husband, 2 daughters, and 1 daughter-in-law)	Content analysis	The service barriers have been identified from family caregivers’ perceptions and there is a need for establishing effective services for meeting the group of people needs
	Focus group discussion		United States, Phoenix			
Vaingankar et al., 2013	Thematic analysis Qualitative study	To develop the informal family caregivers’ experiences, challenges and care needs of caring people with dementia	Home care	63 caregivers (37 adult children, 13 spouses, 13 siblings or grandchildren, or sister/daughter-in-law)	Thematic analysis	The family caregivers experience difficulties and challenges in the care of people with dementia, and there is high demand of supporting of dementia care service, emotion and education
	Focus group discussion, semi-structured interview		Singapore			
Xiao et al., 2013	Gadamer’s philosophical hermeneutics Qualitative study	To explore the family caregivers’ experiences in utilising dementia care services	Home care	13 caregivers (10 adult child, 1 spouse, 1 grandchild, 1 sibling)	Thematic analysis	It implies that the lack of ethno-specific dementia service has a negative impact on utilising and managing for Chinese group family caregivers in dementia care
	Semi-structured interview		Australia			
Koehn et al., 2012	Critical-constructionist theory approach Qualitative study	To develop what sorts of assistance for people with dementia to look for and how support them and caregivers in order to improve the quality of life	Home care	10 people diagnosed Alzheimer’s disease and related dementias (2 women and 8 men), and 11 caregivers (1 husband, 1 daughter, and 8 wives, and 1 son)	Thematic analyses	Exploring the Chinese Canadian family caregivers’ care needs and imply that there is a need for exploring strategies in order to enhance the positive effects of non-pharmacological support
	Semi-structured interview		Canada, Greater Vancouver			
Boughtwood et al., 2011	Grounded theory Qualitative study	To identify caregivers’ perceptions and experiences that take care of family members living with dementia	Home care	121 family carers (37 Chinese, 19 Arabic, 40 Italian, and 25 Spanish)	Thematic analyses	It is important to increase awareness for health professionals and clinicals to provide support dementia care service for family caregivers
	Focus group discussion		Australia			
Netto et al., 2009	Grounded theory Qualitative study	To identify family caregivers’ gained experiences and feelings in the care of people with dementia	Home care	12 participants (8 daughters, 1 spouse, 2 sons, and 1 niece)	Coding paradigm and comparisons	Facilitating the gained experiences and designing dementia activities can be benefit for family caregivers in care of people with dementia
	Semi-structured, face-to-face, In-depth interview		Singapore			
Zhan, 2004	Grounded thery method Qualitative study	To develop Chinese- American caregivers’ experiences of taking care of family member living with dementia	Home setting	4 care recipients and 4 caregivers (3 adult child caregivers, and 1 family relative)	Content analysis	Chinese American caregivers’ feelings regarding caring of family members living with dementia have been investigated, and it is important to reduce stigma to understand them well
	In-depth interview		United Stated			
Ho et al., 2003	Qualitative study	To explore Chinese Canadian caregivers’ stressed experiences regarding taking care of people with Alzheimer’s disease	Home care	12 Chinese- Canadian caregivers (8 daughters, 2 wives, 2 daughters-in-law)	Thematic analyses	It is crucial to provide formal and social service regarding dementia care for meeting family caregivers’ needs with Chinese Canadian culture background
	Semi-structured interview		Canada, Toronto			
Tan et al., 2001	Qualitative study	To explore understanding and emotions of caregivers of taking care of family member living with dementia	Home care	20 caregivers (2 husbands, 1 son-in-law, 4 daughters-in-law, 9 daughters, and 4 wives)	Content analysis	The understanding and experiences of Chinese Australian family caregivers have been identified and synthesised recommendations for the future
	Semi-structured telephone interview		Australia			

### Synthesised Findings

In total, 89 findings were extracted from the 16 included articles. Of those, 88 were ranked as unequivocal, and one was ranked as credible. Based on their similarities, 10 categories were identified. These categories were further conceptualised into four synthesised findings based on the aims of the review (see Online Appendix IV Results of meta-synthesis). These synthesised findings are described as: (1) motivations to take on the caregiving role (categories: Confucianism as motive, Filial piety as a motive); (2) receiving limited dementia care education (categories: lack of dementia care knowledge and skills, desire to learn dementia care); (3) factors affecting access and use of care services (categories: dementia stigma, lack of culturally acceptable care service, embracing ethno-specific care services); and (4) experiencing multifaced challenges (categories: unmanaged changed behaviours, family dynamics, no time to socialise with others). The ConQual summary of findings is presented in Online Appendix V. The synthesised findings and categories are presented in the following sections Munn et al., 2014.

### Synthesised Finding 1: Motivations to Take on the Caregiving Role

#### Filial Piety as a Motive

Adult-child caregivers described their motivation to undertake the caregiving role: ‘It’s an enriching experience and a sense of duty—you bring me up, I look after you. It’s my chance to do a good deed for her (Adult child caregiver)’ (Netto et al., 2009, p. 255). Sharing the caregiver role was widely reported in Chinese families caring for aged parents with dementia: ‘For our Chinese culture, the children look after the parents when they are old. The brothers and sisters have [a] very close relationship. We both love our dad very much and my sister and me … share each other’s burden … (Adult child caregiver)’ (Tan et al., 2001, p. 14). Shared care within the family was perceived as a strategy to reduce caregiver stress:

All of them do appreciate what my youngest sister and I do for my mother and I would say, we are still a very close-knit family. We care for one another and if there is any problem in our midst, we would help out in that sense. I think it has brought us closer (Adult Child caregiver). (Koo et al., 2020, p. 17)

It appeared that shared care in a family was built on strong familial bonding. These cases revealed the influence of filial piety that sustained dementia care at home.

#### Confucianism as a Motive

Perceived duty to a spouse strongly motivated caregivers to overcome challenges in their caregiver role:

The responsibility is mine. I can take care of him like this only because I am his wife. The relationship between husband and wife is the most important. I am the closest to him; I ought to take care of him (Spouse caregiver) (Ho et al., 2003, p. 307).

It was evident in this case that Confucianism had an influence on the spouse caregiver. Such an influence appeared to help spouse caregivers to overcome challenges: ‘Our relationship (giver and receiver) has been good … Sometimes, he makes me very angry, but when I think he used to take care of me, I forgive him (Spouse caregiver)’ (Liu et al., 2020, p. 5).

The findings on motivations for caregivers are evidence that Chinese norms have a profound positive impact on caregivers undertaking their role despite difficulties. The finding supports ‘the principle of life-span development’ in the Life Course Theory that describes cultural influence. The finding that caregivers also demonstrated human agency to improve their practice supports ‘the principle of agency’ described in the Life Course Theory.

### Synthesised Finding 2: Receiving Limited Dementia Care Education

#### Lack of Dementia Care Knowledge and Skills

Caregivers showed a lack of dementia care knowledge and skills necessary for the caregiving role: ‘I did not know why my mom could not find the place where we usually met for lunch (Adult child caregiver)’ (Zhan, 2004, p. 24). They perceived the need to receive educational support at the point of dementia diagnosis: ‘At the beginning, as soon as she is diagnosed with dementia, it’s good to have a relatively good understanding of the condition as well as the kind of care giving that is necessary (Adult child caregiver 12)’ (Tan et al., 2020, p. 264). Caregivers perceived a lack of skills in interacting with their loved one: ‘I didn’t know how to communicate with her, especially in my first year of dementia care (Spouse caregiver)’ (Liu et al., 2020, p. 5). Caregivers also perceived a lack of skills to respond to care recipients who showed changed behaviour: ‘He accuses me of stealing his things. But he hides them and forgets where he had put them. More and more challenges each day (Spouse caregiver)*’* (Tan et al., 2001, p. 13). These findings implied a lack of dementia care education for this cultural group. This is evidence that dementia education for Chinese caregivers is shaped by the historical context in the host country as described in ‘the principle of time and place’ in the Life Course Theory.

#### Desire to Learn Dementia Care

Caregivers expected to access educational support when they needed: ‘I wish there are particular care centres, 24 hours, specialized, those that [can also] train [family] for taking care of dementia patient (Adult child caregiver)’ (Vaingankar et al., 2013, p. 1611). Such a desire may imply the lack of flexibility in providing dementia education and the need to address individualised learning needs through educational support. Again, this finding supports that the availability of dementia care education for this culture group is largely shaped by the historical context in the host country. Peer support was highly valued as part of learning sources:

I learnt a lot of information from the caregiver support group, and when I returned home, I see how to handle things better. Recently I saw my mother’s dental problem, and I was able to know how to handle it from another caregiver (Adult Child caregiver). (Koo et al., 2020, p. 13)

They embraced virtual caregiver support groups using WeChat, a social media application used by the Chinese community: ‘We [Chinese American dementia caregivers] have a WeChat [a Chinese messaging and social media app] group and support each other (Spouse caregiver)’ (Liu et al., 2020, p. 5).

Some caregivers, especially adult child caregivers with English proficiency, accessed the internet to search for information about dementia care services available to them:

*The doctors didn’t tell us anything about dementia care. I didn’t realise there were any services that were directly related to dementia. I learned about dementia care and found the day care service for mum through internet searches (Adult Child caregiver).* (Xiao et al., 2013, p. 5)

The findings on Chinese caregivers’ proactive actions towards gaining information from various learning sources are indicators of human agency as described in ‘the principle of agency’ in the Life Course Theory.

### Synthesised Finding3: Factors Affecting Access and Use of Care Services

#### Dementia Stigma

Caregivers experienced stigma that prevented them from connecting with family members, friends and their local Chinese community by which the sources of help usually available to them were reduced. They described public stigma: ‘They [people in China town] made you feel so ashamed that you are afraid of telling others about you loved one’s illness. It is just so hard (Adult child caregiver)’ (Zhan, 2004, p. 24, p. 24)*.* Caregivers also described self-stigma: ‘My daughter and son-in-law used to take us out to eat, but ever since my husband had dementia [at a very early stage], neither my husband nor I are willing to eat outside (Spouse caregiver)’ (Sun et al., 2014, p. 130).

#### Lack of Culturally Acceptable Care Services

Caregivers perceived that formal care services were mainly designed for those from the mainstream culture. For example, a daughter caregiver was concerned about the loneliness her mother experienced during respite care in a nursing home: ‘She felt very isolated and had no one to talk to. It is better to keep her at home if there is no suitable place for her’ (Adult child caregiver)’ (Xiao et al., 2013, p. 8). The desire for culturally acceptable dementia care services was reinforced by another caregiver: ‘We had to rely on my family members to take care of my mother when I had to go out of town. If there were respite care services that we could trust, I would definitely use them (Adult child caregiver)’ (Sun et al., 2014, p. 129). These cases indicate that caregivers decided not to use formal mainstream care services due to a lack of trust in the social care system. In these given examples, barriers were mainly due to the design of care services for those from the mainstream culture.

#### Embracing Ethno-Specific Care Services

Local Chinese community service organisations were widely used to access dementia and aged care services: ‘The home health agency in Chinatown really helped me a lot; otherwise, I did not know from whom, where, and how I could get help (Adult child caregiver)’ (Zhan, 2004, p. 25). Caregivers embraced Chinese ethno-specific care services as relevant to their culture and language: ‘In the day care, they organised something that mum and dad used to love, namely, watching 1960s–1970s kind of movies. Yeah, they would love things like that and music and sometimes they have karaoke as well (Adult child caregiver)’ (Xiao et al., 2013, p. 8). The findings on barriers to accessing care services were in line with ‘the principle of time and place’ described in the Life Course Theory.

### Synthesised Finding 4: Experiencing Multifaced Challenges

#### Unmanaged Changed Behaviours

Unmanaged behaviours in the person with dementia was widely reported by Chinese diaspora caregivers: ‘He always threw his tantrum and scolded my stepmother and my cousin’s family…His temper was even worse and kicked my cousin out of his house’ (Adult child caregiver)’ (Tan et al., 2001, p. 13). This case indicated that unmanaged behaviours not only caused stress for the primary caregiver, but also the entire family. Chinese diaspora caregivers showed inability to cope with changed behaviour: ‘He accuses me of stealing his things. But he hides them and forgets where he had put them. More and more challenges each day (Wife caregiver)’ (Tan et al., 2001, p. 13).

#### Family Dynamics

Caregivers experienced psychological stress on a daily basis: ‘She [mother-in-law] scolded me nearly every day and I could not bear it anymore. …my husband could not understand and blamed it on me.’ (Daughter-in-law caregiver) (Tan et al., 2001, p. 13). Caregivers also experienced isolation within their own family in their attempt to avoid family conflicts: ‘It [caregiving] has affected my relationship with my children to a certain extent, because I cannot spend time with them…if they come home. He [her father] will get agitated (daughter caregiver)’ (Ho et al., 2003, p. 310). It appeared that lack of support from family members was also identified: ‘My mother lives with me. She cannot speak English and does not like to interact with strangers. They (the brothers) thought it was government’s responsibility to care for Mum. I couldn’t get help from them (Adult child caregiver). (Xiao et al., 2013, p. 6). This case is also evidence that various acculturation levels existed and affected family members’ views about filial piety and the extent to which caregiving approaches were underpinned by this value.

#### No Time to Socialise With Others

Care recipients’ high-level of dependence on caregivers, especially during the night-time, was a source of stress: ‘Not enough sleep every night. Most of the time (feeling) tired (Spouse caregiver)’ (Tuomola et al., 2016, p. 164). The time-dependency on caregivers also contributed to the lack of time to socialise with others and social stress that caregivers perceived: ‘I want to socialize with other people, but now I cannot. I cannot do that because I do not have the time’ (Adult Child caregiver)’ (Ho et al., 2003, p. 310). They perceived: ‘I need the break, away from him [father living with dementia] and I think that helps (Adult child caregiver)’ (Chan et al., 2019, p. 505). These findings indicated that caregivers may lack support within and/or outside of the family.

The findings on the multifaced challenges caregivers experienced supports ‘the principle of life-span development’ described in the Life Course Theory and explains the influence of cultural norms on caregivers’ experiences. Caregivers who hold these norms may choose to take on challenges and sacrifice themselves to the caregiving role.

## Discussion

The use of the Life Course Theory to inform this systematic review contributes to new insights into Chinese diaspora caregivers’ experiences in dementia care. The synthesised findings contribute new knowledge about socially-culturally-historically constructed factors either enabling or hindering Chinese diaspora caregivers’ practices in high-income countries. Our findings also reveal the need to foster cultural adaptation of dementia care education and care services to meet their needs and expectations in dementia care.

Our finding regarding Chinese diaspora caregivers’ motivations to take on their role despite difficulties supports a systematic review that reported a positive association between caregivers’ ethnic minority status in high-income countries and motives to care (Greenwood & Smith, 2019). Although they studied a range of migrant groups, the review identified that the motivations of caregivers from some ethnic groups, including the Chinese, were mainly derived from their cultural norms such as filial piety obligation (Greenwood & Smith, 2019). The positive impact that filial piety has on caregivers may also be explained by the phenomenon that people with dementia living at home are more common in China (95%) than Western Europe (55%) (Alzheimer’s Disease International, 2018). A study of 393 Chinese diaspora caregivers in the US confirmed that a stronger sense of filial piety obligation was associated with a lower level of caregiver burden (Guo et al., 2019). The mechanism underlying such a relationship was explored by Liu et al. (2020) in a study using Grounded Theory. In that study, caregivers who espoused filial piety obligation demonstrated human agency to obtain sources of care from their family, community and the social care system to relieve their stress and learnt from peers to overcome difficulties in daily care activities (Liu et al., 2020).

Our findings that Chinese diaspora caregivers have limited dementia care knowledge and skills reflect the recent World Health Organization (WHO) report highlighting the lack of education and skill training for all caregivers (World Health Organization, 2021). The international community has agreed that ‘75% of countries will provide support and training programmes for carers and families of people with dementia by 2025’ (World Health Organization, 2021, p. 182). However, so far, only 27 out of 77 high-income countries reported providing training programs that were accessible to caregivers (World Health Organization, 2021, p. 191). Moreover, it is not clear whether accessible programs in these 27 countries have been culturally adapted to ethnic minority groups.

We found that Chinese diaspora caregivers desired to learn dementia care at the point of dementia diagnosis and the reason for this was to plan care at an early stage. Our finding supports a previous study which revealed that such support also enabled caregivers to plan care activities that promoted independence for the person with dementia (Kelly & Innes, 2016). Post-diagnosis support for caregivers also has a positive impact on the long-term care plan for care recipients and reduces social isolation for both caregivers and care recipients (Bamford et al., 2021; Kelly & Innes, 2016). To address post-diagnosis support for Chinese diaspora caregivers and other ethnic minority groups, information about dementia care services needs to be provided in the language of their choice and free interpreter services needs to be available in the referral system. Such approaches reflect ‘the principle of time and place’ in the Life Course Theory which emphasises the design of care services for those from ethnic minority groups.

Our finding that dementia stigma is a barrier to Chinese diaspora caregivers seeking help supports previous studies that describe low rates of help seeking by caregivers due to dementia stigma (Nguyen & Li, 2020; Yıkılkan et al., 2014). Our review detailed discrimination towards people with dementia by the Chinese community in high-income countries. A systematic review revealed that when caregivers internalised public stigma they were more likely to experience lower self-esteem, negative emotional reactions and depression (Nguyen & Li, 2020). In addition, dementia stigma also reinforces misunderstandings of dementia as a ‘madness’ or untreatable; therefore, family caregivers are unwilling to seek help from health professionals or use social care services (Nguyen & Li, 2020). Participation in dementia-related education is associated with significant stigma reduction (Herrmann et al., 2018), suggesting that culturally adapted dementia care education is much needed.

Our finding on the lack of culturally acceptable care services as a reason for Chinese diaspora caregivers to withdraw from, or not to use, services support previous studies in Australia and UK (Baghirathan et al., 2020; Brijnath et al., 2021). Such situations were attributed to caregivers’ perception that mainstream care services could ‘diminish’ their loved ones due to the lack of culturally and linguistically congruent care (Baghirathan et al., 2020; Czapka & Sagbakken, 2020). It is evident that bilingual persons with dementia who spoke fluently in the dominant language of a particular country prior to dementia usually regress to their first language. Therefore, expecting them to adapt to mainstream care services is not realistic and contributes to inequalities in dementia care between groups in high-income countries (Martin et al., 2018; Xiao et al., 2021). Our review, along with other studies, indicate that ethno-specific aged care providers are more likely to address inequalities in dementia care by providing culturally adapted care services, employing bilingual and bicultural staff, and building trust with ethnic communities (Baghirathan et al., 2020; Brijnath et al., 2021).

Our finding on unmanaged changed behaviours and the negative impact of this on Chinese diaspora caregivers (i.e., caregiver stress and distress) is in line with a systematic review of the general population of people with dementia in the community setting (Feast et al., 2016). However, it is well recognised that causes and triggers contributing to changed behaviours differ from case to case and consequently require individualised coaching and counselling services (Cheng et al., 2020). Our finding revealed that interventions need to allow Chinese diaspora caregivers to use their language of choice to explore causes and triggers as this will empower them to develop a care plan to manage changed behaviours based on their situation. Having trained bilingual and bicultural health professionals to work with them showed effectiveness (Ma & Saw, 2020; Xiao et al., 2016).

Our findings detailed multifaced challenges experienced by Chinese diaspora caregivers which were rarely reported in studies specific to this group of caregivers. The finding that Chinese diaspora caregivers experienced stress due to the lack of support from family members reflects a systematic review on caregivers in general (Oh et al., 2020), but reasons attributed to the situation may differ between ethnic groups. In general, reasons attributed to the situation include the expectation for females in the family to perform the caregiving role and avoidance of unacceptable behaviours in the person with dementia (Oh et al., 2020). Our review supports these reasons but pointed out that family members who adapted well to the mainstream culture may believe less in filial piety, be less likely to share in care and may add stress to the primary caregiver due to deferent attitudes and views towards caregiving.

### Strengths and Limitations

This review showed a number of strengths. First, there were no restrictions placed regarding the publication date of articles reviewed. Therefore, findings are based on all studies in the study field. Second, the JBI meta-aggregated approach and tools enabled the reviewers to demonstrate a transparent process for extracting data, grouping original findings and synthesising findings based on the research question. This review also has limitations. First, the included studies were published in the English language only. The possibility of omitting publications in non-English language journals and language bias in data searched exists. Second, a search of grey literature was not conducted in this review. Therefore, this review does not include studies reported in the grey literature.

## Conclusion

The use of Life Course Theory to interpret findings from studies on Chinese diaspora caregivers has implications for policy, resource and care service development in dementia care. First, government policies in dementia care design dementia care service should support the establishment and/or advancement of ethno-specific dementia care services to meet the caregivers’ expectations for culturally and linguistically congruent care for people with dementia from ethnic minority groups. Second, government needs to invest in culturally adapted psychoeducation to address the gap in education interventions for this group of caregivers. Third, care service providers need to engage Chinese diaspora caregivers in care planning and care activities taking into account of the impact of filial piety and Confucianism on their motivation to take on the caregiving role. Fourth, dementia care service providers need to adapt their services to meet this caregiver group’s needs, preferences and expectations. Fifth, to address multifaced challenges, individualised coaching and counselling services for caregivers need to be available and accessible. In addition, considering the various acculturation levels of family members, dementia care education needs to target the family as a unit in order to support the primary caregiver.

## Supplemental Material

Supplemental Material - Chinese diaspora caregivers’ experiences in dementia care in high-income countries: A systematic review Click here for additional data file. Supplemental Material for Chinese diaspora caregivers’ experiences in dementia care in high-income countries: A systematic review by Yujing Zhang, Lily Xiao, and Jing Wang in Dementia
