# Supplementary material for: Chinese diaspora caregivers’ experiences in dementia care in high-income countries: A systematic review
Source: Dementia (London). 2023 Apr 18;22(5):1115–37. doi: 10.1177/14713012231169830 (PMC10262336; doi:10.1177/14713012231169830)
Supplement: Supplemental Material - Chinese diaspora caregivers’ experiences in dementia care in high-income countries: A systematic review [file sj-pdf-1-dem-10.1177_14713012231169830.pdf]

List of Appendix

Appendix I Keywords and search terms……………………………………………………….2

Appendix II Searched databases………………………………………………………….3

Appendix III Extraction of findings from included studies………………………………..7

Appendix IV Results of meta-synthesis…………………………………………………....21

Appendix V ConQual summary of findings………………………………………………..24

Appendix VI Recommendations for practice……………………………………………...26

Appendix I Keywords and search terms

Family caregiver	Experience	Dementia	Chinese
home caregiver<br>informal caregiver<br>unpaid caregiver<br>unprofessional caregiver<br>caring	feeling<br>perception<br>emotion<br>care need<br>challenge<br>care burden	Alzheimer's disease<br>cognitive impairment	China

Appendix II Searched databases

Following the search strategies, six English databases are used for searching qualitative studies or mixed-methods studies with qualitative content regarding Chinese caregivers' experiences in the care of family members living with dementia. The searched results of each database are presented in the column.

CINAHL

#	Search terms	Results
S1	TI (("family caregiver*" or "home caregiver*" or "informal caregiver*" or "unpaid caregiver*" or "unprofessional caregiver*" or caring*)) OR AB (("family caregiver*" or "home caregiver*" or "informal caregiver*" or "unpaid caregiver*" or "unprofessional caregiver*" or caring*))	55,057
S2	MH "family caregiver*" OR MH "home caregiver*" OR MH "informal caregiver*" OR MH "unpaid caregiver*" OR MH "unprofessional caregiver*" OR MH caring*	9,022
S3	S1 OR S2	59,517
S4	TI ((experience* or feeling* or perception* or emotion* or challenge* or "care need*" or "care burden*")) OR AB ((experience* or feeling* or perception* or emotion* or challenge* or "care need*" or "care burden*"))	862,042
S5	MH experience* OR MH feeling* OR MH perception* OR MH emotion* OR MH challenge* OR MH "care need*" OR MH "care burden*"	73,641
S6	S4 OR S5	885,116
S7	TI (dementia or alzheimers or cognitive impairment or memory loss) OR AB ((dementia or Alzheimer* or "cognitive impairment"))	101,980
S8	MH dementia OR MH Alzheimer* OR MH "cognitive impairment"	75,503
S9	S7 OR S8	117,501
S10	TI ((Chinese or China)) OR AB ((Chinese or China))	95,987
S11	MH Chinese OR MH China	63,690

S12	S10 OR S11	113,310
S13	S3 AND S6 AND S9 AND S12	65

EMcare

Query	Search terms	Results
1	caregiver/	57,857
2	"care and caring"/	326
3	("family caregiver*" or "home caregiver*" or "informal caregiver*" or "unpaid caregiver*" or "unprofessional caregiver*" or caring*).tw,kw.	44,470
4	1 or 2 or 3	89,778
5	experience/	8,503
6	emotion/	41,520
7	perception/	95,451
8	health care need/	20,483
9	caregiver burden/	5,888
10	(experience* or feeling* or perception* or emotion* or challenge* or "care need*" or "care burden*").tw,kw.	1,028,933
11	5 or 6 or 7 or 8 or 9 or 10	1,060,484
12	dementia/	54,324
13	Alzheimer disease/	55,136
14	cognitive defect/	55,629
15	(dementia or Alzheimer* or "cognitive impairment").tw,kw.	116,861
16	12 or 13 or 14 or 15	159,349
17	Chinese/	19,671
18	China/	57,887
19	(Chinese or China).tw,kw.	128,088
20	17 or 18 or 19	139,421
21	4 and 11 and 16 and 20	146

Medline

#	Search terms	Results
1	Caregivers/	45,672
2	("family caregiver*" or "home caregiver*" or "informal caregiver*" or "unpaid caregiver*" or "unprofessional caregiver*" or caring*).tw,kf.	58,596
3	1 or 2	91,038
4	Emotions/	78,057
5	Perception/	41,370
6	Caregiver Burden/	405
7	(experience* or feeling* or perception* or emotion* or challenge* or "care need*" or "care burden*").tw,kf.	2,413,294
8	4 or 5 or 6 or 7	2,441,965
9	Dementia/	58,124
10	Alzheimer Disease/	109,761
11	Cognitive Dysfunction/	30,084
12	(dementia or Alzheimer* or "cognitive impairment").tw,kf.	290,559
13	9 or 10 or 11 or 12	316,134
14	China/	226,560
15	(Chinese or China).tw,kf.	474,019
16	14 or 15	521,761
17	3 and 8 and 13 and 16	114

ProQuest

#	Search terms	results
S1	noft(("family caregiver*" OR "home caregiver*" OR "informal caregiver*" OR "unpaid caregiver*" OR "unprofessional caregiver*" OR caring*)) AND noft((experience* OR feeling* OR perception* OR emotion* OR challenge* OR "care need*" OR "care burden*")) AND noft((dementia OR Alzheimer* OR "cognitive impairment")) AND noft((Chinese OR China))	93

Scopus

#	Search terms	results
S1	(TITLE-ABS-KEY (("family caregiver*" OR "home caregiver*" OR "informal caregiver*" OR "unpaid caregiver*" OR "unprofessional caregiver*" OR caring*)) AND TITLE-ABS-KEY ((experience* OR feeling* OR perception* OR emotion* OR challenge* OR "care need*" OR "care burden*")) AND TITLE-ABS-KEY ((dementia OR alzheimer* OR "cognitive impairment")) AND TITLE-ABS-KEY ((chinese OR china)))	97

Web of Science

#	Search terms	results
S1	TOPIC: ((“family caregiver*” OR “home caregiver*” OR “informal caregiver*” OR “unpaid caregiver*” OR “unprofessional caregiver*” OR caring*)) AND TOPIC: ((experience* OR feeling* OR perception* OR emotion* OR challenge* OR “care need*” OR “care burden*”)) AND TOPIC: ((dementia OR Alzheimer* OR “cognitive impairment”)) AND TOPIC: ((Chinese OR China))	164

Appendix III Extraction of findings from included studies

Note: C= Adult child caregiver; S=Spouse caregiver; R= Relative family caregiver

Koo, M. Y., Pusey, H., & Keady, J. (2020). 'I try my best ... I try to relieve the burden of my mum': a narrative analysis of the everyday care-giving experiences for five intergenerational Singapore-Chinese families where one member has dementia. *Ageing and Society* 1-23. doi:10.1017/S0144686X20000070

Findings	Illustration from study	Evidence
Peer support as a source of learning (C)	"I learnt a lot of information from the caregiver support group and when I returned home, I see how to handle things better. Recently I saw my mother's dental problem and I was able to know how to handle it from another caregiver. (Sixth interview)" (p.13)	Unequivocal
Family bond as a source of dementia care (C)	"All of them do appreciate what my youngest sister and I do for my mother and I would say, we are still a very close-knit family. We care for one another and if there is any problem in our midst, we would help out in that sense. I think it has brought us closer. (Sixth interview)" (p.17)	Unequivocal

Liu, J., Lou, Y., Wu, B., & Mui, A. C. Y. S. (2020). "I've been always strong to conquer any suffering:" challenges and resilience of Chinese American dementia caregivers in a life course perspective. *Aging and Mental Health,* 1-9. doi:10.1080/13607863.2020.1793900

Findings	Illustration from study	Evidence
Difficulty in identifying bilingual professionals	"It is very troublesome to take her to see a doctor, so I want to find a bilingual doctor who could make house calls. It is very difficult to	Unequivocal

(C)	find one. (66 years old, daughter who provided care for her mother)" (p.5)	
Reluctance to gaining service due to dementia stigma (C)	"Her children [caregiver's siblings-in-law] don't want to apply for any benefits for her [care receiver] because they don't want others to know their mom has dementia. They are concerned that no one will marry to their kids [care receivers' grandchildren] because the disease may be inherited. (55 years old, daughter-in-law who provided care for her mother-in-law)" (P.5)	Unequivocal
Virtual caregiver support group as a source of learning (S)	"We [Chinese American dementia caregivers] have a WeChat [a Chinese messaging and social media app] group and support each other. (76 years old, wife who provided care for her husband)" (P.5)	Unequivocal
Language barrier in applying for services (S)	"My English is not good. It is a huge burden for me to fill out forms or pay for bills. (71 years old, wife who provided care for her husband)" (P.5)	Unequivocal
Sacrifice retirement life to caregiver role (S)	"I used to think that my post-retirement life would be beautiful, such as traveling and volunteering. Now I can't do anything. (80 years old, husband who provided care for his wife)" (P.5)	Unequivocal
Reduced traditional family support from children (caused by culture adaptation) (S)	"My son-in-law is an ABC [America-born Chinese]. He doesn't like living with older people, especially after my husband has dementia. (76 years old, wife who provided care for her husband)" (P.5)	Unequivocal
Reciprocity as a motive for caregiver (S)	"Our relationship [giver and receiver] has been good. … Sometimes he makes me very angry, but when I think he used to take care of me, I forgive him. (71 years old, wife who provided care for her husband)" (P.5)	Unequivocal
Lack of knowledge about communication with the care recipient (S)	"When I didn't understand dementia, I didn't know how to communicate with her, especially in my first year of dementia care. (89 years old, husband who provided care for his wife)" (P.5)	Unequivocal
Emotional stress (caused by care arrangement) (S)	"What worries me most is that I may die before her. If that happens, who will take care of her? My children will have a huge burden. (86 years old, husband who provided care for his wife)" (P.5)	Unequivocal
Physical exhaustion (C)	"Tired, very tired. … I can't sleep well. … Very stressful. (58 years old, daughter who provided care for her father)" (p.5)	Unequivocal

Self-care by physical exercise<br>(S)	"I insist on exercising at home every day. Before he [care receiver] wakes up, I have some time to do it. If my health is poor, how can I take care of him? (71 years old, wife who provided care for her husband)" (P.5)	Unequivocal

Tan, L. L., Ong, P. S., Ng, L. L., Ng, W. F., Wong, H. K., & Sim, A. C. C. (2020). Decision-Making in dementia care: A qualitative study of Chinese family caregivers in Singapore. *Annals Academy of Medicine Singapore*, *49*(4), 263-267. Retrieved from <Go to ISI>://WOS:000533623700011

Findings	Illustration from study	Evidence
Keeping the care recipients informed of dementia diagnosis<br>(C)	"At least get her mentally prepared. At least she can face, we don't need to hide from her … so she will accept. At least prepare and accept the facts that there will be some difficulty in handling her living style here and there. (Caregiver 7)" (p.264)	Unequivocal
Lack of knowledge about dementia care at early stage<br>(C)	"At the beginning, as soon as she is diagnosed with dementia, it's good to have a relatively good understanding of the condition as well as the kind of care giving that is necessary. (Caregiver 12)" (p.264)	Credible

Chan, E. Y., Phang, K. N., Glass, G. F., & Lim, W. S. (2019). Crossing, trudging and settling: A phenomenological inquiry into lived experience of Asian family caregivers of older persons with dementia. *Geriatric nursing 40*(5). doi:10.1016/j.gerinurse.2019.03.015

Findings	Illustration from study	Evidence
Caregiving around the clock<br>(C)	"Then after that when she [mother living with dementia]is resting, I have to run to the market come back and cook...school time I have to rush, send my son, then later fetch him. It's like a daily job. (P4, daughter)" (p.504)	Unequivocal
Coping strategy for feeding<br>(C)	"She (care recipient) said, 'No, no, no. she (domestic helper) wants to poison me cannot.' ...So, I take the same plate, I bring it to the	Unequivocal

	kitchen. I don't let her see...So I just turn one round, I come back and say, ‘I cook the rice for you...’ She said okay. Then she will finish her meal. (P10, daughter)” (p.505)	
Self-care by taking a break<br>(C)	“If I need a break, I will go out for a walk or go out with my friends. I need the break, away from him and I think that helps. That's how I find my way to get along. (P3, son)” (p.505)	Unequivocal
Positive attitude towards caregiver role<br>(C)	“If he deteriorates and has to go to nursing home, we will accept it as it we know this is inevitable. When my father is still around, I show love to him. Even if he passes on the next day, I will not regret. (P13, daughter)” (p.506)	Unequivocal
Spirituality as a motive for caregiving<br>(S)	“Every day, when I have finished my tasks, I must have quiet time for myself...I will use the time to pray. (p.13, daughter)” (p.506)	Unequivocal

Lun, M. W. A. (2019). Chinese American family caregivers' perception of program use and caregiver stress. *Journal of Social Service Research, 45*(5), 750-758. doi:10.1080/01488376.2018.1514679

Findings	Illustration from study	Evidence
Emotional burden attributed to caregiver’s poor health<br>(S)	“Of course, I felt more irritated … blamed myself for not having enough strength to take care of my wife … If my health is better, I can take a better care of her. Whenever I feel weak, I feel more irritated and madder. (A husband caregiver)” (p.754)	Unequivocal
Insufficient information about dementia care service<br>(C)	“I am curious of what resources are out there … any support groups, group programs, government programs that I can look into … A lot of this we do not know much … resources. (A daughter caregiver)” (p. 755)	Unequivocal

Tuomola, J., Soon, J., Fisher, P., & Yap, P. (2016). Lived experience of caregivers of persons with dementia and the impact on their sense of self: A qualitative study in Singapore. *Journal of Cross-Cultural Gerontology, 31*(2), 157-172. doi:10.1007/s10823-016-9287-z

Findings	Illustration from study	Evidence
Emotional burden (multiple care responsibilities related)<br>(S)	"I have to take care of myself, home, and children when they come every week, got to think of what meals to give them. There are a lot of things; I am like a house keeper. (P6)." (p.163)	Unequivocal
Physical burden (lack of sleep)<br>(S)	"Not enough sleep every night. Most of the time [feeling] tired (P6)." (p.164)	Unequivocal
Emotional burden attributed to care recipients' memory loss<br>(S)	"Whatever I can tell him at night, I will talk [to] him, he is like before, he will [nods head]. But next morning it is gone. It is a complete wipe-out (P1)." (p.164)	Unequivocal
Acceptance of caregiver role<br>(S)	"Just face it that he is like that, it does not affect me, I am ok. I understand that he is like that, not that he does it on purpose, that he cannot remember (P2)." (p.165)	Unequivocal
Obligation for the care recipient<br>(S)	"What to do, that is your husband, you must take care of him (P4)" (p.163)	Unequivocal
Reciprocity as a motive for the caregiver role<br>(S)	"So now, I have to do everything that he used to do for me, just like payback time (P1)" (p.166)	Unequivocal
Positive self-appraisal<br>(S)	"I have become more patient, [and] exercise more self-control, which I am still learning. I feel that [going] through a harder life tends to make you a better person. This is the hard way of learning about life (P5)" (p.163)	Unequivocal

Caldwell, L., Low, L.-F., & Brodaty, H. (2014). Caregivers' experience of the decision-making process for placing a person with dementia into a nursing home: comparing caregivers from Chinese ethnic minority with those from English-speaking backgrounds. *International Psychogeriatrics, 26*(3), 413-424. doi:10.1017/S1041610213002020

Findings	Illustration from study	Evidence
Perceived dilemma when deciding to use nursing home (C)	"Late last year I decided to put my mum in the queue. But I actually don't want her to go even if a position is available … but end up you know we say "Ok we put her on the queue." Just in case … Because we understand she's already 86, things may drop at any time. (CW2)" (p.417)	Unequivocal
Responsibility as a motive for home care (C)	"If I put my mum in the nursing home, I'm the bad guy … I fail my duty (CW5)." (P419)	Unequivocal
Negative thoughts about nursing home care (C)	"It's just too early stage to take her to the nursing home. Reason being that one, going to nursing home is just like going to jail … (CW14)" (p.418)	Unequivocal

Sun, F., Mutlu, A., & Coon, D. (2014). Service barriers faced by Chinese American families with a dementia relative: Perspectives from family caregivers and service professionals. *Clinical Gerontologist, 37*(2), 120-138. doi:10.1080/07317115.2013.868848

Findings	Illustration from study	Evidence
Lack of bilingual professional caregivers to relieve family caregivers (C)	"We wanted to look for a home taker [formal caregiver] for my mother, but we could not find bilingual and skilled formal caretakers (A daughter caregiver)" (p.129)	Unequivocal
Lack of ethno-specific nursing homes (Chinese culture related) (C)	"We wanted to look for a home taker [formal caregiver] for my mother, but there are few residential care facilities for Chinese patients. (A daughter caregiver)" (p.129)	Unequivocal
Lack of information about dementia care services (S)	"I don't know whether there are any supportive services available in the community and nobody told us. (A spousal caregiver)" (p.129)	Unequivocal
Desire for respite care service (C)	"We had to rely on my family members to take care of my mother when I had to go out of town. If there were respite care services that	Unequivocal

	we could trust, I would definitely use them (A daughter caregiver).” (p.129)	
Self- stigma towards dementia (S)	“My daughter and son-in-law used to take us out to eat, but ever since my husband had dementia [at very early stage], neither my husband nor I are willing to eat outside (A spouse caregiver).” (p.130)	Unequivocal

Vaingankar, J., Subramaniam, M., Picco, L., Eng, G., Shafie, S., Sambasivam, R., . . . Chong, S. (2013). Perceived unmet needs of informal caregivers of people with dementia in Singapore. *International Psychogeriatrics, 25*(10), 1605-1619. doi:10.1017/S1041610213001051

Findings	Illustration from study	Evidence
Need for respite care (C)	“I think caregivers recognize there’s a need for, what they call respite care. They (should) take leave to care for themselves, take a break, short holiday or just rest. (P5FE002)” (p.1609)	Unequivocal
Emotional stress due to memory loss of care recipients (C)	“The fear of someone very dear to you will one day not recognize who you are, things that caregiver will encounter, mainly in the first stage . . . at that time we do not know how to handle it because we fear, we keep thinking, one day if my mom not recognize (us), how are we going to handle that part? (P5FE002)” (p.1608)	Unequivocal
Lack of post-diagnosis support from medical doctors (C)	“The problem lies with the doctor . . . they can’t tell you exactly what to do. When I asked the doctor, he said “you need to notice yourself.” He said that my mother’s condition would get worse after six to nine months, but he did not tell me how to deal with it either. (P12SIC002)” (p.1609)	Unequivocal
The need for social support service (C)	“What type of services we need, like support centres for dementia or other types of care centres – who will be able to help us or give us counselling on how to handle this kind of emotional (problem). (P6FE002)” (p.1609)	Unequivocal
Emotional burden attributed to cost on dementia care (C)	“I have to work. My wife is not working, I have two children, so the only solution I have for my dad right now is nursing home or long-term stay. But they are expensive. (P6FE003)” (p.1610)	Unequivocal

Lack of appropriate facilities to care for people with sever BPSD (C)	"I had admitted him to a nursing home three days ago; yesterday they called me and said that they had to throw him out, because he was caught with problems. And he even molested the staff there, so they threw him back (to the hospital). (P6FE003)" (p.1610)	Unequivocal
Lack of dementia-friendly outpatient clinics services (C)	"It's always a very long waiting time, my dad cannot control his bowels and he got angry very fast . . . I tell them (clinic staff), please help me to let him go first . . . Then my dad starts to get angry, very angry and that's when everybody starts to look at us . . . when my dad finally threw a tantrum, then they let my dad go first. (P10FT001)" (p. 1610)	Unequivocal
Expectation for outpatient clinics to provide dementia-friendly services (C)	"I want a hospital to be more understanding for the dementia patient, reduce our waiting time. (P10FT001)" (p. 1610)	Unequivocal
The need to provide dementia education at the care centre (C)	"I wish there are particular care centres, 24 hours, specialized, those that (can also) train (family) for taking care of dementia patient. (P3FC003)" (p.1611)	Unequivocal

Xiao, L. D., De Bellis, A., Habel, L., & Kyriazopoulos, H. (2013). The experiences of culturally and linguistically diverse family caregivers in utilising dementia services in Australia. *BMC Health Services Research*, *13*(1), 427-438. https://doi.org/10.1186/1472-6963-13-427

Findings	Illustration from study	Evidence
Language barrier to accessing formal dementia care services (C)	"She felt very isolated and had no one to talk to. It is better to keep her at home if there is no suitable place for her' [daughter caregiver]." (p. 8)	Unequivocal
Chinese ethno-specify care services (C)	"In the day care, they organised something that mum and dad used to love, namely, watching 1960s-1970s kind of movies. Yeah, they would love things like that and music and sometimes they have karaoke as well' [Daughter caregiver]" (p. 8.)	Unequivocal

Accessed internet to search information (C)	"The doctors didn't tell us anything about dementia care. I didn't realise there were any services that were directly related to dementia. I learned about dementia care and found the day care service for mum through internet searches [Daughter caregiver]" (p. 5)	Unequivocal
Reduced traditional shared care by children (caused by cultural adaptation) (C)	"Although our culture expects that the first son will take care of the elderly parents, my older brother never did that for my parents. He and my other brothers only visit my parents occasionally. My mother lives with me. She cannot speak English and does not like to interact with strangers. They [the brothers] thought it was government's responsibility to care for Mum. I couldn't get help from them [Chinese 12]." (Xiao et al., 2013). (p. 6)	Unequivocal

Koehn, S., McCleary, L., Garcia, L., Spence, M., Jarvis, P., & Drummond, N. (2012). Understanding Chinese–Canadian pathways to a diagnosis of dementia through a critical-constructionist lens. *Journal of Aging Studies, 26*(1), 44-54. doi:10.1016/j.jaging.2011.07.002

Findings	Illustration from study	Evidence
Emotional burden attributed to untreated BPSD (S)	"I noticed not only memory impairment, but also [his] personality changed. He became suspicious and [had] delusion[s], he told me our house has another person. Actually, only I and he lived here. Sometime, he suspected I have a boyfriend and gossiped to his friends (Judy)." (p.48)	Unequivocal
Using online information about dementia online (S)	"I read Readers' Digest, it has a topic related with dementia and it provides the website. I kicked into their Internet and requested for further information. It sent me the information. It introduced ten signs and symptoms of dementia, I checked and my husband has eight (Judy)." (p.48)	Unequivocal
Lack of information about social service (C)	"I did not know [about support services]. I don't know many people here. I did not come across these social services and their information before (Ping)." (p.50)	Unequivocal
Support from social workers (referral support service related) (C)	"It was after referral that the social worker contacted us themselves and told me that they have these services and visits that could help me to see if they could provide me with some information or limited services to help me to see how to take care of my mother together...	Unequivocal

	[Now] there are people who could tell me because before I am totally blank with these concepts (Ping).” (p.50)	

Boughtwood, D. L., Adams, J., Shanley, C., Santalucia, Y., & Kyriazopoulos, H. (2011). Experiences and perceptions of culturally and linguistically diverse family carers of people with dementia. *American Journal of Alzheimer's Disease and Other Dementias®, 26*(4), 290-297. doi:10.1177/1533317511411908

Findings	Illustration from study	Evidence
Physical strain attributed to lack of suitable equipment at home<br>(S)	“She is big and fat, I can’t lift her up. What I usually do is pull over all of the chairs we have. Then I gently put her onto the shortest chair, and from there, put her onto a higher chair, and then the highest chair to help get her up. My back becomes extremely sore after all that (Chinese family carer, husband)” (p.293)	Unequivocal
Inability to manage care recipient’s BPSD<br>(C)	“I can only look after him for two or three hours because after that, my father starts to look for my mother. He would ask me where my mother has gone to and say that he wants her back. He is used to my mother (Chinese family carer, son)” (p.294)	Unequivocal

Netto, N. R., Jenny, G. Y. N., & Philip, Y. L. K. (2009). Growing and gaining through caring for a loved one with dementia. *Dementia, 8*(2), 245-261. doi:10.1177/1471301209103269

Findings	Illustration from study	Evidence
Be patient with the care recipient<br>(C)	“Because of her, I train myself to be more patient. throughout the years, I think I have trained up myself to be more patient. But I think the patience level have to rise further in order to better deal with her. (Mrs J)” (p.250)	Unequivocal
Focusing on close relationship with care recipient<br>(S)	“I’m drawn closer to him. There’s that closeness causes I pay so much attention to him, I understand all his needs, so learning to love him would be better. (Mrs L)” (p.254)	Unequivocal
Improved family relationship via caregiver role<br>(C)	“I think because of this caregiving experience, we have become closer. We realize that we treasure our loved ones more and we understand that unity is very important in the family when things	Unequivocal

	happen like that. The bonding of the family, united as well as be each other's support. (Mrs H)" (p.254)	
Filial piety as a motive for caregivers (C)	"It's an enriching experience and a sense of duty – you bring me up, I look after you. It's my chance to do a good deed for her. (Mr E)" (p.255)	Unequivocal

Zhan, L. (2004). Caring for family members with Alzheimer's Disease: Perspectives from Chinese American caregivers. *Journal of gerontological nursing, 30*(8), 19-29. doi:10.3928/0098-9134-20040801-06

Findings	Illustration from study	Evidence
Lack of knowledge about dementia (C)	"I did not know she had AD. We only knew when the doctor told us that my mom needed help." (p.23)	Unequivocal
Lack of knowledge about initial dementia signs (C)	"I did not know why my mom could not find the place where we usually met for lunch." (p.24)	Unequivocal
Public stigma towards dementia (C)	"They [people in China town] made you feel so ashamed that you are afraid of telling others about you loved one's illness. It is just so hard." (p.24)	Unequivocal
Lack of post-diagnosis support from health professionals (C)	"I knew it was a bad diagnosis. I was very upset that they [health providers] did not provide more support and information. You cannot just tell the diagnosis and walk away." (p.25)	Unequivocal
Inability to speak English as a barrier to accessing nursing home (C)	"I was looking for a long-term care facility for my mom. Staff at the facility told me that they would not take my mom because she did not speak English." (p.25)	Unequivocal
Using ethno-specific dementia care service (C)	"The home health agency in Chinatown really helped me a lot; otherwise, I did not know from whom, where, and how I could get help." (p. 25)	Unequivocal
Learning to be a caregiver via caregiver support group (C)	"I joined the AD support group. I got information and learned about how to find resources, how to handle the patient but not feel frustrated and not to irritate patients because you are stressed or burned out, and how to take care of ourselves." (p.25)	Unequivocal

The need to provide dementia care education Chinese<br>(C)	"We need to educate the Chinese community about AD so that people can try to help one another rather than walk away when we need support." (p.26)	Unequivocal
Sacrificing personal life to caregiver role<br>(C)	"Even though I sacrificed my personal life, I had no regret for caring for my mom." (p.26)	Unequivocal

Ho, B., Friedland, J., Rappolt, S., & Noh, S. (2003). Caregiving for relatives with Alzheimer's disease: Feelings of Chinese-Canadian women. *Journal of Aging Studies, 17*(3), 301-321. doi:10.1016/S0890-4065%2803%2900028-8

Findings	Illustration from study	Evidence
Family relationship as a motive for caregivers<br>(S)	"The responsibility is mine. I can take care of him like this only because I am his wife. The relationship between husband and wife is the most important. I am the closest to him; I ought to take care of him. (A wife caregiver)" (p.307)	Unequivocal
Filial piety as a motive for caregivers<br><br>(C)	"I think the part of our Chinese culture, that one has to take care of and respect elderly people, is right. I think it really is culture... being filial to our parents is right; take care of them is what we ought to do. We ought to live with them and hope that we can give even more than what they are getting now. (A daughter caregiver)" (p.308)	Unequivocal
Lack of time to socialise with others<br>(C)	"I do not have time to socialize with others. I want to socialize with other people, but now I cannot. I cannot do that because I do not have the time. (A daughter-in-law caregiver)" (p.310)	Unequivocal
Role conflict within a family (C)	"It has affected my relationship with my children to a certain extent, because I cannot spend time with them. It is the same if they come home. He [her father] will get agitated. (A daughter caregiver)" (p.310)	Unequivocal
Time-dependence burden<br>(C)	"I should expand my social life outside … after she [her mother] got Alzheimer's disease, my life has been very limited. My life is limited to the home. I rarely interact with other people (A daughter caregiver)." (p. 311)	Unequivocal

Positive thoughts on caregiver's role (C)	"I often say that my mother is like my flower. If I cherish her well, I will be very happy. I have this feeling that I will be very happy if she is well because I have put in my effort. I do not mean that she has to give whatever back to me, the bottom line is that I did all that because I wanted her to be healthy. (A daughter caregiver)" (p.312)	Unequivocal
Family support for the primary caregiver (C)	"If my family did not support me like this, I would not be able to hang in for so long, I would have placed him [her father] in a nursing home a long time ago. (A daughter caregiver)" (p.313)	Unequivocal
Reduced burden by using government-subsidised home care programs (C)	"I am so thankful for the government; it has helped me a lot. He [the home care worker] helps him with the shower, and also does the house cleaning for us, so my burden is not as heavy. (One daughter caregiver)" (p.314)	Unequivocal
Supported from social workers (C)	"[The social workers] were supportive in a lot of things and have given me a lot of support. Although I was having a difficult time, having these people give me support for sure made me happier. (A daughter caregiver)" (p.314)	Unequivocal

Tan, L., Fleming, A., & Ledwidge, H. (2001). The caregiving burden of relatives with dementia: experiences of Chinese-Australian families. *Geriaction, 19*(1), 10-16. Retrieved from https://search.informit.com.au/search;res=IELHEA;search=FTI=yes%20AND%20IS=1032-4410%20AND%20VRF=19%20AND%20IRF=1%20AND%20PY=2001%20AND%20PG=10

Findings	Illustration from study	Evidence
Disturbance in sleep due to BPSD (S)	"I now sleep in another room because he gets up so many times in the night. (Wife)" (p.11)	Unequivocal
Maintaining safety for the care recipient with BPSD (S)	I have to make sure all the doors are locked and keep the keys on me or he would go out at night. (Wife)" (p.11)	Unequivocal
Shared caregiver responsibilities within	"We like to keep everything within the family. We share our	Unequivocal

the family<br>(C)	responsibilities and we do not like to "wash dirty linen in public." We must also keep our family honour and respect all elders. (Daughter)" (p.12)	
Inability to manage continence issues<br>(C)	"He went to the toilet at night-time all the time and wet the floor and the toilet bowl. He was not incontinent but when he went into the toilet, his pants were partly wet already. (Daughter)" (p.12)	Unequivocal
Difficulty in managing care recipients' BPSD (C)	"She started having urinary incontinence. It was getting harder in looking after her. (Daughter-in-law)" (p.12)	Unequivocal
Inability to manage BPSD<br>(S)	"He accuses me of stealing his things. But he hides them and forgets where he had put them. More and more challenges each day. (Wife)." (p.13)	Unequivocal
Psychological stress associated to unmanaged BPSD<br>(C)	"He always threw his tantrum and scolded my stepmother and my cousin's family. He insisted to cook for himself and always made a big mess. His temper was even worse and kicked my cousin out of his house. (Daughter)" (p.13)	Unequivocal
Family conflicts attributed to caregiver role<br>(C)	"As I had to stay with her all the time, my husband and me had a lot of arguments. I treated my children badly because my temper was bad too. (Daughter-in-law)" (p.14)	Unequivocal
Psychological stress (BPSD and family related)<br>(C)	"She [mother-in-law] scolded me nearly every day and I could not bear it any more. At first my husband could not understand and blamed it on me. I stayed away from home for a few weeks and I hoped the situation would get better. When I returned home, my mother-in-law has not changed. She still scolded me. (Daughter-in-law)" (p.13)	Unequivocal
Shared caregiver role with family members<br>(C)	"For our Chinese culture, the children look after the parents when they are old. The brothers and sisters have very close relationship. We both love our dad very much and my sister and me ... share each other's burden.(Daughter)" (p.14)	Unequivocal
Support from children for the primary caregiver<br>(S)	"All my children pay for everything their mother needs and they take turns to come here to look after her. They even pay for my holidays so that I can take a break. (Husband)" (p.14)	Unequivocal

Appendix IV Results of meta-synthesis

<table>
<tr><th>Findings</th><th>Categories</th><th>Synthesised findings</th></tr>
<tr><td>Filial piety as a motive for caregivers(U)</td><td rowspan="5">Filial piety as a motive</td><td rowspan="25">Synthesised finding 1: Motivations to take on the caregiving role
Caregivers who held filial piety and Confucianism values were motivated to undertake the caregiving role and overcome challenges.</td></tr>
<tr><td>Filial piety as a motive for caregivers (U)</td></tr>
<tr><td>Sacrificing personal life to caregiver role (U)</td></tr>
<tr><td>Shared caregiver role with family members (U)</td></tr>
<tr><td>Family bond as a source of dementia care (U)</td></tr>
<tr><td>Reciprocity as a motive for the caregiver role (U)</td><td rowspan="20">Confucianism as a motive</td></tr>
<tr><td>Reciprocity as a motive for a spouse caregiver (U)</td></tr>
<tr><td>Family relationship as a motive for caregivers (U)</td></tr>
<tr><td>Responsibility as a motive for home care (U)</td></tr>
<tr><td>Obligation for the care recipient (U)</td></tr>
<tr><td>Spirituality as a motive for caregiving (U)</td></tr>
<tr><td>Family support for the primary caregiver (U)</td></tr>
<tr><td>Support from children for the primary caregiver (U)</td></tr>
<tr><td>Shared caregiver responsibilities within the family (U)</td></tr>
<tr><td>Improved family relationship via caregiver role (U)</td></tr>
<tr><td>Positive attitude towards caregiver role (U)</td></tr>
<tr><td>Positive thoughts on caregiver’s role (U)</td></tr>
<tr><td>Acceptance of caregiver role (U)</td></tr>
<tr><td>Positive self-appraisal (U)</td></tr>
<tr><td>Keeping the care recipients informed of dementia diagnosis (U)</td></tr>
<tr><td>Coping strategy for feeding (U)</td></tr>
<tr><td>Maintaining safety for the care recipient with BPSD (U)</td></tr>
<tr><td>Being patient with the care recipient (U)</td></tr>
<tr><td>Focusing on close relationships with care recipients (U)</td></tr>
<tr><td>Self-care by physical exercise (U)</td></tr>
<tr><td>Lack of knowledge about initial dementia signs (U)</td><td rowspan="4">Lack of dementia care knowledge and skills</td><td rowspan="4">Synthesised finding 2: Receiving limited dementia care education
Caregivers showed a lack of dementia care knowledge and skills necessary for the caregiving role.</td></tr>
<tr><td>Lack of knowledge about dementia (U)</td></tr>
<tr><td>Lack of knowledge about communication with the care recipient (U)</td></tr>
<tr><td>Inability to manage BPSD (U)</td></tr>
</table>

Difficulty in managing care recipients' BPSD (U)		They perceived the need to receive educational support at the point of dementia diagnosis. They expected to access educational support when they needed. They highly valued peer support including virtual caregiver support groups using a social media application.
Inability to manage continence issues (U)		
Lack of knowledge about dementia care at early stage (C)		
Limited opportunity for providing dementia care education in Chinese community (U)		
Lack of information about dementia care services (U)		
Insufficient information about dementia care service (U)		
Lack of information about social service (U)		
Lack of post-diagnosis support from medical doctors (U)		
Lack of post-diagnosis support from health professionals (U)		
Expectation for outpatient clinics to provide dementia-friendly services (U)		
Expectation for respite care (U)		
Learning to be a caregiver via caregiver support group (U)	Desire to learn dementia care	
Peer support as a source of learning (U)		
Virtual caregiver support group as a source of learning (U)		
Using online information to inform dementia care (U)		
Accessed internet to search information (U)		
Desire of satisfising individualised dementia care learning needs (U)		
Reluctance to gaining service due to dementia stigma (U)	Dementia stigma	**Synthesised finding 3: Factors affecting access and use of care services** Caregivers experienced stigma that prevented them from connecting with family members, friends and their local Chinese community by which the sources of help usually available to them were reduced. Caregivers perceived that formal care services were mainly designed for those from the mainstream
Public stigma towards dementia (U)		
Self-stigma towards dementia (U)		
Inability to speak English as a barrier to accessing nursing home (U)	Lack of culturally acceptable care service	
Lack of bilingual professional caregivers to relieve family caregivers (U)		
Language barrier in applying for services (U)		
Difficulty in identifying bilingual professionals (U)		
Lack of ethno-specific nursing homes (Chinese culture related) (U)		
Language barrier to accessing formal dementia care services (U)		

Lack of acceptable dementia care service (U)		culture. Caregivers embraced Chinese ethno-specific care services as relevant to their culture and language.
Lack of dementia-friendly outpatient clinics services (U)		
Negative thoughts about nursing home care (U)		
Lack of appropriate facilities to care for people with sever BPSD (U)		
Perceived dilemma when deciding to use nursing home (U)		
The need for social support service (U)	Embracing ethno-specific care services	
Using ethno-specific dementia care service (U)		
Chinese ethno-specify care services (U)		
Supported from social workers (U)		
Support from social workers (referral support service related) (U)		
Reduced burden by using government-subsidised home care programs (U)		
Emotional burden attributed to untreated BPSD (U)	Unmanaged changed behaviours	**Synthesised finding 4: Experiencing multifaced challenges**<br>Unmanaged behaviours in the person with dementia was widely reported by caregivers. Caregivers showed inability to cope with changed behaviour. Caregivers also experienced family conflicts relating to their caregiving role. They were unable to socialise with others.
Emotional burden attributed to care recipients' memory loss (U)		
Emotional burden attributed to caregiver's poor health (U)		
Emotional burden (multiple care responsibilities related) (U)		
Emotional stress due to memory loss of care recipients (U)		
Emotional burden attributed to cost on dementia care (U)		
Emotional stress (caused by care arrangement) (U)		
Psychological stress (BPSD related) (U)		
Psychological stress associated to unmanaged BPSD (U)		
Inability to manage care recipient's BPSD (U)		
Reduced traditional family support from children (caused by cultural adaptation) (U)	Family dynamics	
Family conflicts attributed to caregiver role (U)		
Unmanaged changed behaviours contributed to role conflict within a family(U)		
Psychological stress (BPSD and family related) (C)		
Physical exhaustion (U)	No time to socialise with others	
Physical burden (lack of sleep) (U)		
Disturbance in sleep due to BPSD (U)		

Physical strain attributed to lack of suitable equipment at home (U)		
Caregiving around the clock (U)		
Lack of time to socialise with others (U)		
Time-dependence burden (U)		
Sacrifice retirement life to caregiver role (U)		
High-level dependence on caregivers (U)		

Appendix V ConQual summary of findings

**Systematic review title:** Chinese diaspora caregivers' experiences in dementia care in high-income countries: A systematic review<br>**Population:** Chinese caregivers from high-income countries<br>**Phenomena of interest:** Caregivers' experiences, emotions, expectations, feelings and perspectives in dementia care<br>**Context:** Home care				
**Synthesized finding**	**Type of research**	**Dependability**	**Credibility**	**ConQual score**
**Synthesised finding 1: Motivations to take on the caregiving role**<br>Caregivers who held filial piety and Confucianism values were motivated to undertake the caregiving role and overcome caregiving challenges.	Qualitative	High	High	Moderate
**Synthesised finding 2: Receiving limited dementia care education**<br>Caregivers showed a lack of dementia care knowledge and skills necessary for the caregiving role. They perceived the need to receive educational support at the point of dementia diagnosis. They expected to access educational support when they needed. They highly valued peer support including virtual caregiver support groups using a social media application.	Qualitative	High	Downgrade 1 level**	Moderate
**Synthesised finding 3: Factors affecting access and use of care services**<br>Caregivers experienced stigma that prevented them from connecting with family members, friends and their local Chinese community by which the sources of help usually available to them were reduced. Caregivers perceived that formal care services were mainly designed for those from the mainstream culture. Caregivers embraced Chinese ethno-specific care services as relevant to their culture and language.	Qualitative	High	High	Moderate

**Synthesised finding 4: Experiencing multifaced challenges** Unmanaged behaviours in the person with dementia was widely reported by caregivers. Caregivers showed inability to cope with changed behaviour. Caregivers also experienced family conflicts relating to their caregiving role. They were unable to socialise with others.	Qualitative	High	High	Moderate

**The credibility downgrade one level due to a mix of unequivocal and credible findings

Appendix VI Recommendations for practice

Recommendations	JBI Grade
Government policies in dementia care should support the establishment and/or advancement of ethno-specific dementia care services to meet the caregivers' expectations for culturally and linguistically congruent care for people with dementia from ethnic minority groups.	A
Government needs to investment in culturally adapted psychoeducation to address the gap in education interventions for this group of caregivers.	A
Care service providers need to engage Chinese diaspora caregivers in care planning considering the impact of filial piety and Confucianism on their motivation to take on the caregiving role.	A
Dementia care service providers need to culturally adapt their services to meet this caregiver group's needs, preferences, and expectations.	A
To address multifaced challenges, individualised coaching and counselling services for Chinese caregivers need to be available and accessible for this group of caregivers.	A
Considering the various acculturation levels of family members, dementia care education needs to target the family as a unit in order to support the primary caregiver.	A
